# Piezocatalytic ZnS: Mn^2+^ Nanocrystals for Enhanced Organic Dye Degradation

**DOI:** 10.53941/mi.2024.100005

**Published:** 2024-11-21

**Authors:** Zhongxiang Wang, Elizaveta Tiukalova, Youyi Tai, Miaofang Chi, Jin Nam, Yadong Yin

**Affiliations:** 1 Department of Chemistry, University of California, Riverside, CA 92521, USA; 2 Center for Nanophase Materials Sciences, Oak Ridge National Laboratory, Oak Ridge, TN 37831, USA; 3 Department of Bioengineering, University of California, Riverside, CA 92521, USA

**Keywords:** piezocatalysis, piezoelectricity, zinc sulfide, pollutant degradation, water decontamination

## Abstract

Piezocatalysis, an emerging approach that harnesses mechanical energy to drive chemical reactions, has garnered significant attention due to its potential applications in diverse fields, particularly in environmental remediation. Its broader application, however, is often hindered by the low efficiency of existing piezocatalytic materials. Here, we report the synthesis of Mn^2+^-doped ZnS nanocrystals with improved piezoelectric properties using an emulsion-based colloidal assembly technique. Through well-controlled Mn^2+^ doping, these nanocrystals demonstrate high piezocatalytic activity for degrading organic dyes under ultrasonic vibration. The optimal performance is achieved with 3% Mn^2+^ doping, outperforming many existing piezocatalysts. Mechanistic studies reveal the generation of reactive oxygen species as the primary driving force for degradation. Notably, pre-excitation with UV light further boosts the piezocatalytic efficiency of these metal ion-doped ZnS nanocrystals by filling electron trap states, leading to improved overall performance. This research paves the way for developing high-performance piezocatalysts, expanding the potential of piezocatalysis for a wide range of applications.

## Graphical Abstract

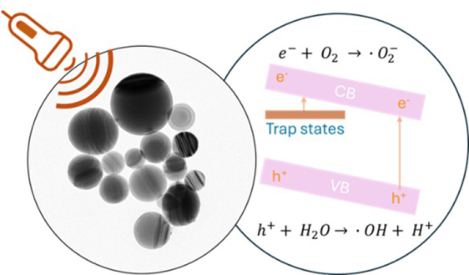

## 1. Introduction

The rapid industrial development has led to a surge in water-soluble organic pollutants, posing severe environmental and health concerns. Traditional water treatment methods often fall short of effectiveness in addressing these pollutants, necessitating the exploration of advanced oxidation processes (AOPs). Coupled with photocatalysis [1–3] electrocatalysis [4,5] and Fenton reactions [6–8] these methods have emerged as promising decontamination strategies due to their ability to generate reactive radicals for breaking down a wide range of organic molecules into less harmful substances. Photocatalysis utilizes light to activate semiconductor materials, creating electron-hole pairs to drive radical formation. Similarly, electrocatalysis applies an electrical current to induce redox processes, while Fenton reactions use iron-based catalysts to break down hydrogen peroxide into highly reactive hydroxyl radicals. Despite their effectiveness, these processes also face their own challenges, such as significant energy inputs or dependence on specific reagents, prompting the need for developing alternative catalytic strategies.

Recently, the piezoelectric effect has been explored to use mechanical energy to drive degradation reactions of organic pollutants [9,10], leading to the development of a new catalytic process termed piezocatalysis. Piezocatalysis distinguishes itself from traditional electrocatalysis and photocatalysis by utilizing charge separation in piezoelectric materials when subjected to mechanical vibrations [11,12]. This unique mechanism enables a range of chemical reactions, making piezocatalysis an attractive option for environmental remediation [13–17], energy sustainability [18–20], and biomedical applications [21–23]. Commonly studied piezocatalytic materials include ZnO and BaTiO_3_, known for their non-centrosymmetric crystal structures and piezoelectric coefficients ranging between 20 and 100 pC/N [23,24]. However, these materials face limitations such as instability in acidic environments or potential ecological and cytotoxic concerns [25,26]. Alternatively, wurtzite-phase zinc sulfide (ZnS), another non-centrosymmetric piezoelectric material with good chemical stability and biocompatibility, has shown decent piezocatalytic performance in hydrogen generation from pure water [18]. Nonetheless, due to its lower piezoelectric coefficient than ZnO and BaTiO_3_, ZnS has been less explored for piezocatalytic applications. Moreover, its transition from the stable zinc blende to the more piezoelectrically active wurtzite phase typically requires high-temperature calcination [27,28], which, however, increases its bulkiness, making it difficult to compete with nanoscale catalysts featuring extremely high specific surface area. Therefore, strategies to enhance the piezoelectric properties of ZnS nanocrystals and expand their use in piezocatalysis are of significant research interest.

In this study, we report the synthesis of manganese-doped ZnS (ZnS:Mn^2+^) nanocrystals from their quantum dot precursors using an emulsion-based assembly approach. The resulting nanocrystals exhibited exceptionally high piezoelectricity, with a piezoelectric coefficient of up to 23 pC/N, and outstanding piezocatalytic efficiency for the degradation of model organic pollutants, including methylene blue (MB) and rhodamine B (RhB), under ultrasound vibration. Our mechanistic studies revealed that the degradation process was driven by hydroxyl and superoxide radicals generated from charge carriers under sonication. Notably, UV pre-excitation of the catalysts significantly enhanced the piezocatalytic performance by filling the trap states and promoting charge separation during sonication. The enhanced piezoelectricity and piezocatalytic activity can be attributed to the synergistic effects of doping-induced point defects and assembly-caused stacking faults within the nanocrystals, with the former creating trap states that facilitate charge separation and the latter enhancing spontaneous polarization. These material characteristics promotes the generation and utilization of charge carriers under mechanical vibration, leading to improved catalytic performance. This innovative synthesis approach to develop high-performance piezocatalytic materials opens new avenues for sustainable environmental remediation and biomedical applications.

## 2. Results and Discussion

**Figure 1.**
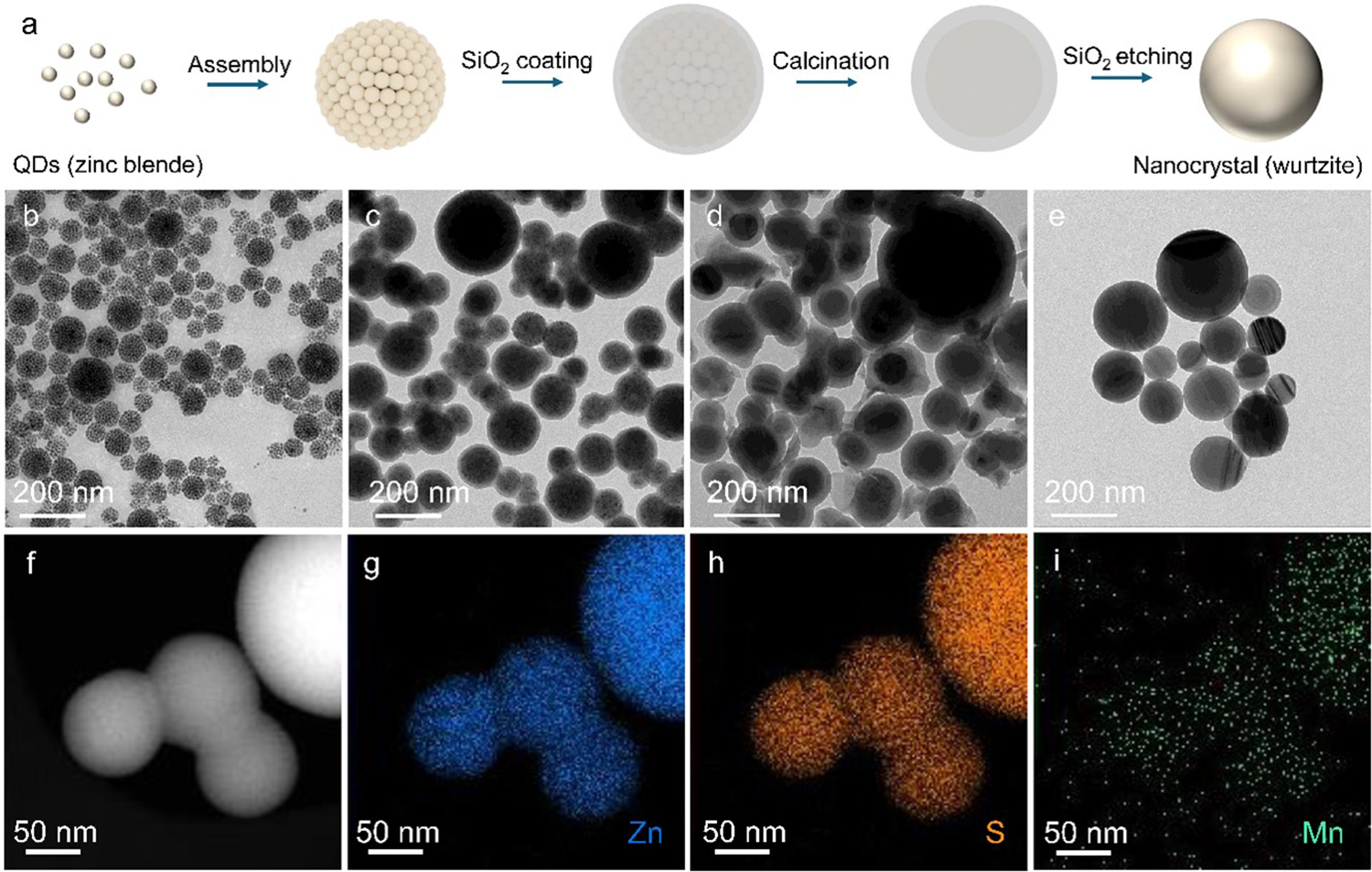
(**a**) Fabrication process of ZnS-based nanocrystals. (**b**–**e**) TEM images of clusters of ZnS:3%Mn^2+^ QDs (**b**), silica-coated clusters of ZnS:3%Mn^2+^ QDs (**c**), silica-coated ZnS:3%Mn^2+^ nanocrystals after calcination (**d**), and the released ZnS:3%Mn^2+^ nanocrystals after removing silica via base etching (**e**). (**f**) HAADF-STEM image of ZnS:3%Mn^2+^ nanocrystals. (**g**–**i**) EDS mapping of Zn (**g**), S (**h**), and Mn (**i**) in ZnS:3%Mn^2+^ nanocrystals.

Figure 1a depicts the process of synthesizing wurtzite phase ZnS nanocrystals, using their zinc blende phase quantum dots (QD) as precursors. It begins with dispersing pre-synthesized QDs in droplets of an oil-in-water emulsion, allowing the QDs to form clusters as the solvent evaporates, and then coating the clusters with a layer of silica. Subsequent steps involve calcination of the core-shell particles, during which QD clusters transform into wurtzite nanocrystals and sinter into larger domains, followed by selective etching of the silica using sodium hydroxide (NaOH) to release the wurtzite nanocrystals. Specifically, pure ZnS QDs and ZnS:Mn^2+^ QDs were synthesized at a high temperature (300 °C) via a Lewis acid-base reaction between metal chlorides and elemental sulfur, with oleylamine as the solvent and capping ligand [29,30]. The synthesis produced QDs with a relatively narrow size distribution, as evidenced in the transmission electron microscopy (TEM, Thermo Fisher Scientific, Waltham, MA, USA) image in Figure S1a for a sample of 3% Mn^2+^-doped ZnS QDs with an average diameter of approximately 9.4 nm (Figure S1b). The Mn^2+^ doping level does not appear to affect the QD size, as indicated by the TEM images of undoped (Figure S1c) and 1% Mn^2+^-doped ZnS QDs (Figure S1d). Using cetyltrimethylammonium bromide (CTAB) as the surfactant, the QD solution in cyclohexane was dispersed in water to produce an oil-in-water emulsion. Subsequent evaporation of cyclohexane promoted the assembly of QDs into clusters [31]. The detailed procedures are provided in the Supporting Information. Figure 1b displays the TEM image of QD clusters with an average size of ~81 nm (size distribution shown in Figure S2). Since the as-prepared QDs are in the zinc blende phase [30], high-temperature calcination is required to induce the phase transition from zinc blende to wurtzite as the latter is more piezoelectrically active due to the lack of inversion symmetry. To prevent sintering between the clusters during calcination, they were coated with a silica layer using the Stöber method [32]. Figure 1c presents the TEM image of silica-coated clusters, which have an average coating thickness of ~11.5 nm, estimated based on 30 measurements (Figure S3). The silica-coated clusters were then calcined at 1050 °C for 90 min to convert them into larger nanocrystals (Figure 1d) and induce the zinc blende-to-wurtzite phase transition. After the silica layer was removed by NaOH etching, spherical nanocrystals in the wurtzite phase were obtained, as shown in Figures 1e and S4a. The average size of these nanocrystals was determined to be ~76 nm, based on the statistical size distribution presented in Figure S4b. These nanocrystals were further characterized using aberration-corrected scanning transmission electron microscopy (STEM, JEOL, Freising, Germany) equipped with energy-dispersive X-ray spectroscopy (EDS, JEOL, Freising, Germany). Figure 1f provides the high-angle annular dark-field (HAADF) STEM image of ZnS:3%Mn^2+^ nanocrystals, while Figure 1g–i show that Mn^2+^ was successfully incorporated into the final nanocrystals through initial doping during QD synthesis, with Zn, S, and Mn elements uniformly distributed throughout the nanocrystals.

**Figure 2.**
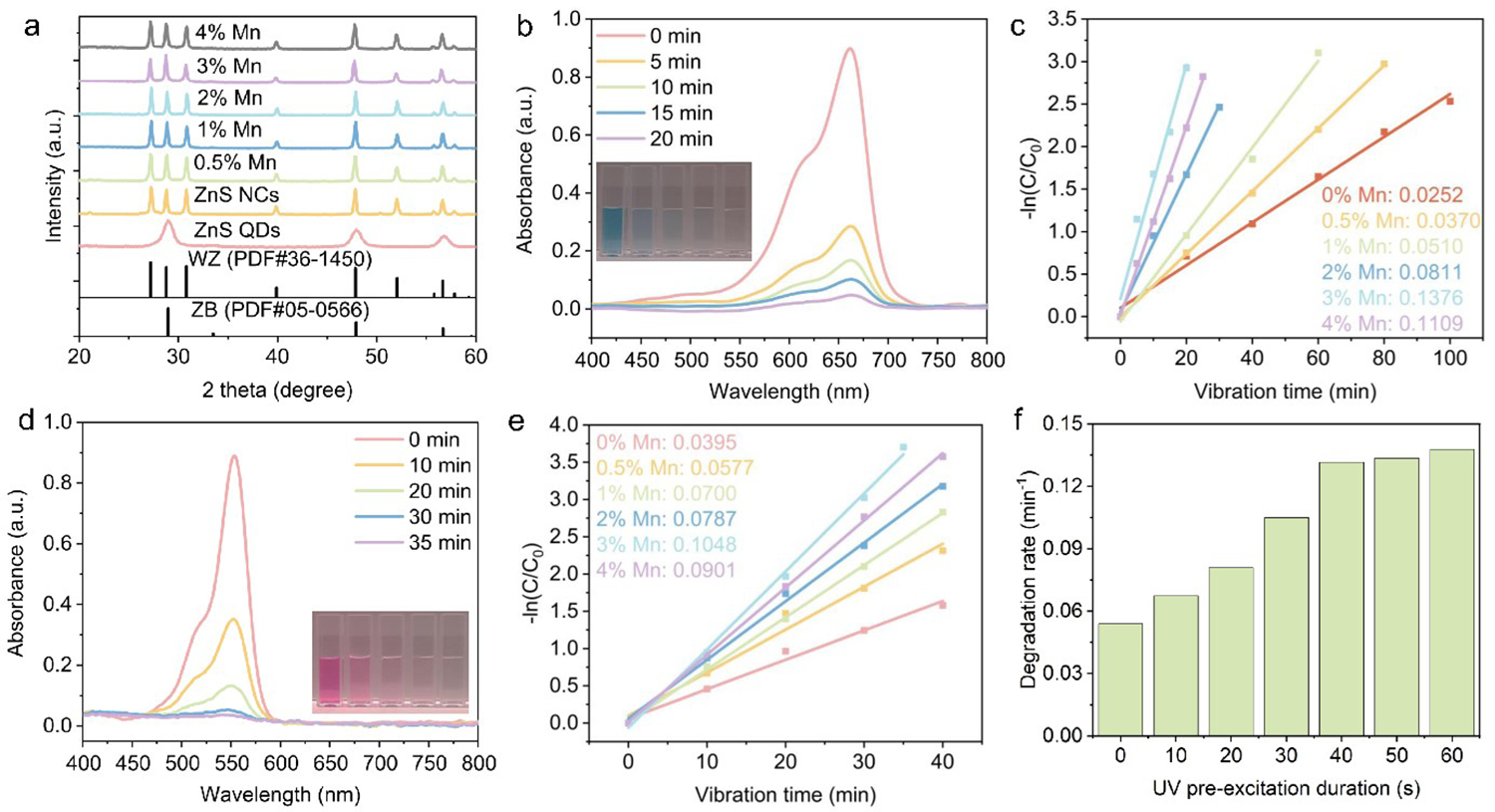
(**a**) XRD patterns of the ZnS QDs precursors and ZnS nanocrystals synthesized by calcination. The nanocrystals were doped with varying amounts of Mn^2+^. (**b**,**d**) UV-Vis spectra of MB (**b**) and RhB (**d**) after piezocatalytic degradation by ZnS:3%Mn^2+^ nanocatalysts for varying periods. (**c**,**e**) Piezocatalytic performance of ZnS:Mn^2+^ nanocatalysts for degrading MB (**c**) and RhB (**e**). (**f**) Piezocatalytic degradation rates of MB using ZnS:3%Mn^2+^ nanocatalysts pre-excited under UV for varying durations.

Figure 2a shows the X-ray diffraction (XRD, PANalytical, Almelo, The Netherlands) patterns of the ZnS QD precursors and the ZnS nanocrystals doped with varying amounts of Mn^2+^ (0%–4%). The ZnS QD precursors exhibit a zinc blende phase, displaying peaks consistent with the standard powder diffraction file (PDF#05–0566). After assembly and calcination, the nanocrystals transit to a wurtzite phase, as confirmed by comparing to the standard PDF#36–1450, regardless of the Mn^2+^ doping concentration. When the Mn^2+^ doping ratio exceeds 3%, a slight peak shift to lower angles is observed. This can be attributed to the larger ionic radius of Mn^2+^ (0.83 Å) compared to Zn^2+^ (0.74 Å) [33], which increases the lattice parameter and shifts the diffraction peaks to lower angles according to Bragg’s Law. To investigate the piezoelectric properties, piezoresponse force microscopy (PFM, Asylum Research, Goleta, CA, USA) was performed on the ZnS:3%Mn^2+^ wurtzite nanocrystals. Figure S5 displays the response amplitude (A) of the nanocrystals under a voltage bias (B) of 3 V, resulting in a piezoelectric coefficient (d_33_) of 23.3 pm/V, calculated using the equation *d*_*33*_ = *A·f/(B·Q*_*f*_*)*, where *f* is the correction factor and *Q*_*f*_ is the quality factor (*Q*_*f*_ = 150) [28]. This high d_33_ value indicates strong piezoelectricity in the nanocrystals, significantly exceeding previously reported values for ZnS [34].

The piezoelectric ZnS-based nanocrystals were utilized as catalysts for dye degradation. The catalyst loading was maintained at 1 mg/mL, and the initial dye concentration was set to 10 ppm (or mg/L). The catalysts were stirred with the dyes in the dark to achieve adsorption-desorption equilibrium. Figure S6a shows the UV-Vis spectra of methylene blue (MB) during the stirring process with the catalysts for up to 60 min. The corresponding plot of C/C_0_ over time, derived from Beer’s Law (Figure S6b), indicates that 30 min of stirring is sufficient to establish adsorption-desorption equilibrium.

In the subsequent step, the mixture was exposed to UV light with emission centered at 365 nm for 1 min to fill the trap states within the bandgap of ZnS [35]. It was observed that exposure to UV light did not alter the dye concentration (Figure S7), confirming that no photocatalytic degradation occurred during this step. Subsequently, the mixture was subjected to ultrasound treatment at 40 kHz while maintaining a constant temperature of 22 °C. Aliquots were taken at specific time intervals, and the nanocrystals were separated by centrifugation. Figure 2b displays the UV-Vis spectra of MB degradation catalyzed by ZnS:3%Mn^2+^ nanocrystals. The MB was rapidly degraded under sonication, achieving 95% degradation within just 20 min. The degradation ratio (D) was calculated by:

(1) $$D=\left(1-\frac{C}{C_{0}}\right)\times 100\%=\left(1-\frac{A}{A_{0}}\right)\times 100\%$$

where *C* is the concentration at a given time during the reaction, *C*_0_ is the initial concentration, *A* is the absorbance at a given time during the reaction, and *A*_0_ is the initial absorbance value [16]. The inset photos in Figure 2b depict the color change of the MB solution during the piezocatalytic degradation process. The degradation of dyes follows a simplified Langmuir-Hinshelwood first-order kinetics model:

(2) $$-\text{ln}\left(\frac{C}{C_{0}}\right)=-\text{ln}\left(\frac{A}{A_{0}}\right)=kt$$

where *k* represents the degradation rate constant.

ZnS nanocrystals with varying Mn^2+^ doping ratios were tested for their efficiencies in degrading MB. Figure S8 shows the UV-Vis spectra of MB degradation using ZnS nanocrystals doped with different Mn^2+^ ratios (0%, 0.5%, 1%, 2%, 3%, and 4%). The degradation rate constants, calculated using Equation (2), are summarized in Figure 2c. The ZnS nanocrystals doped with 3% Mn^2+^ exhibited the fastest degradation, with the highest rate constant of 0.1376 min^−1^. To further confirm the piezocatalytic performance of the ZnS-based nanocrystals, Rhodamine B (RhB) with an initial concentration of 10 ppm was also used as a model organic dye. Figure 2d presents the UV-Vis spectra of RhB degradation catalyzed by ZnS:3%Mn^2+^ over 35 min, with the inset showing the color change of the RhB solution at various stages of degradation. The degradation ratio of RhB reached 95% after 30 min of sonication, as calculated using Equation (1).

Figure S9 shows the UV-Vis spectra of RhB degraded by ZnS:Mn^2+^ nanocrystals with doping ratios ranging from 0% to 4%. The degradation rate constants, determined using Equation (2), are summarized in Figure 2e. Again, the ZnS nanocrystals doped with 3% Mn^2+^ exhibited the highest degradation rate constant of 0.1048 min^−1^. Control experiments were conducted by sonicating the organic dyes in the absence of catalysts. Figure S10a,b displays the UV-Vis spectra of MB and RhB during 40 min of sonication. The absorbances of both dyes showed no significant decrease, indicating that sonication alone is insufficient to degrade the dye molecules, and the observed degradation is primarily due to the catalytic activity of the ZnS-based nanocrystals.

The enhancement of piezocatalytic degradation efficiency by UV pre-excitation, a unique feature of our piezocatalysts, was further investigated using ZnS:3%Mn^2+^ nanocatalysts. The UV pre-excitation process involves exposing the dye/nanocatalyst mixture to UV light before initiating sonication, and it is believed to modify the electronic states of the nanocrystals. As illustrated in Figure S11, the degradation of MB was markedly slower when the pre-excitation step was omitted. Specifically, the MB degradation rate was calculated to be 0.0539 min^−1^ without UV pre-excitation, compared to a significantly higher rate of 0.1397 min^−1^ with UV pre-excitation of 1 min. These findings highlight the crucial role of UV pre-excitation in achieving efficient piezocatalytic degradation of organic dyes.

The observed enhancement in the degradation rate by UV pre-excitation can be attributed to the ability of UV light to excite electrons within the ZnS:Mn^2+^ nanocrystals, filling the trap states that are otherwise empty.^24^ These trapped charge carriers can then be readily released under mechanical vibrations during the sonication process, participating in redox reactions that degrade the dye molecules. This mechanism underscores the importance of pre-activating the catalyst to maximize the utilization of charge carriers during the piezocatalytic process.

To further substantiate this hypothesis, we explored the effect of varying the UV pre-excitation time, ranging from 10 s to 1 min, on the degradation rates of MB. The results, presented in Figure 2f, demonstrate that the degradation rate initially increased with the duration of UV pre-excitation, reaching an optimal rate after 40 s. Beyond this point, the degradation rate plateaued, suggesting that the photoexcited electrons fully occupied the trap states, and additional UV exposure did not contribute to further improvements. The UV-Vis absorption spectra of MB degradation under different pre-excitation times are shown in Figure S12. The data clearly demonstrate that UV pre-excitation is essential for optimizing the piezocatalytic performance of the ZnS:Mn^2+^ nanocrystals. The lack of further noticeable enhancement in degradation rates beyond 40 s of pre-excitation likely indicates the saturation of electron trap states. This saturation behavior aligns with the hypothesis that UV pre-excitation maximizes the availability of reactive charge carriers, thereby boosting the overall efficiency of the piezocatalytic degradation process.

**Figure 3.**
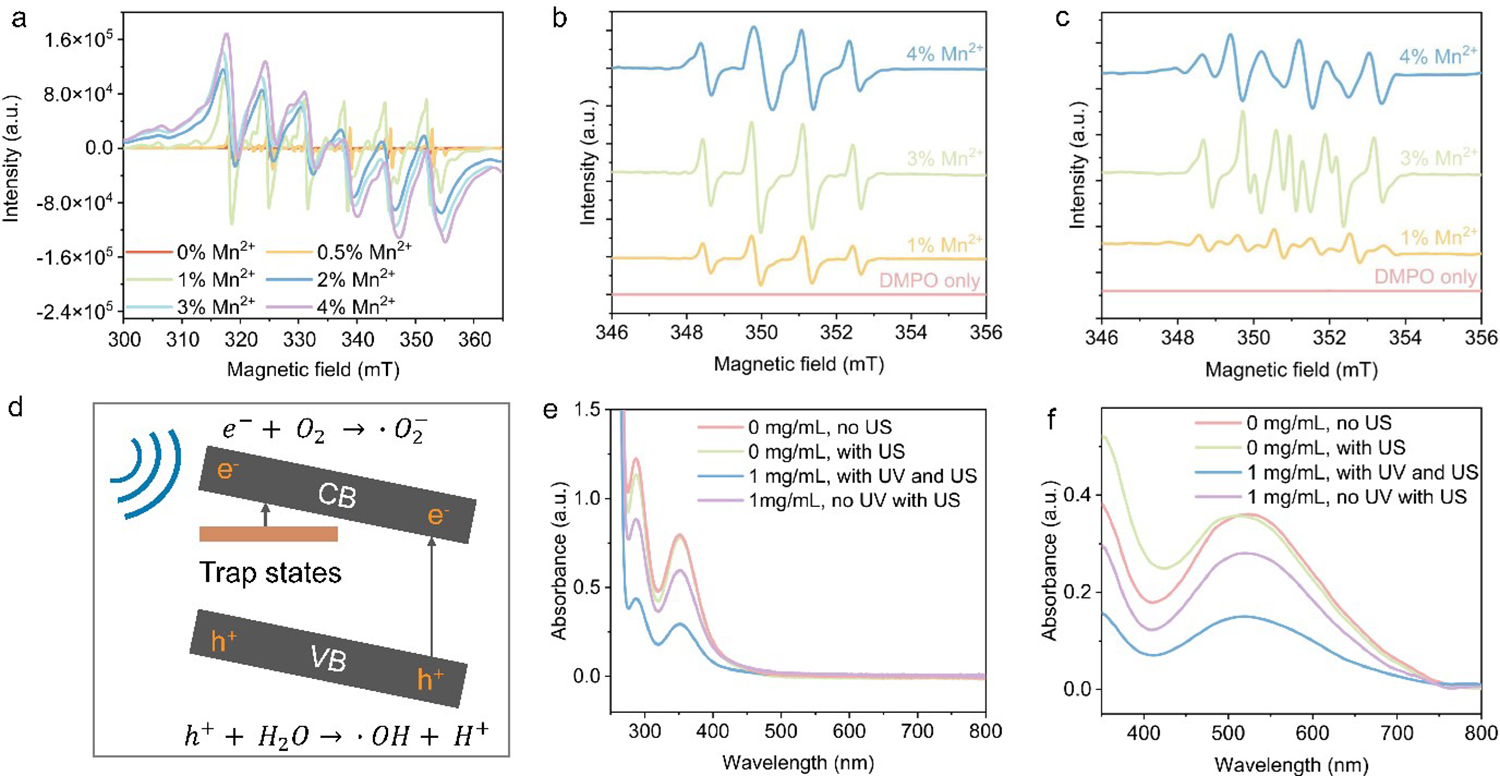
(**a**) EPR spectra of ZnS nanocrystals doped with different amounts of Mn^2+^. (**b**) EPR spectra of DMPO-trapped hydroxyl radicals in water generated from sonication of water-dispersed ZnS:Mn^2+^ nanocrystals with 1%, 3%, and 4% doping, compared with DMPO/water solution. (**c**) EPR spectra of DMPO-trapped superoxide radicals in water generated from sonication of water-dispersed ZnS:Mn^2+^ nanocrystals with 1%, 3%, and 4% doping, compared with DMPO/water solution. (**d**) Scheme of charge separation for ZnS:Mn^2+^ nanocrystals under sonication. (**e**,**f**) UV-Vis spectra of $I_{3}^{-}$ (**e**) and oxidized SA (**f**) generated during the reaction under various conditions including with or without ZnS:3%Mn^2+^ catalyst (labeled with concentration), UV pre-excitation (labeled as “UV”), and (ultra)sonication (labeled as “US”).

To elucidate the effect of Mn^2+^ doping ratios on piezocatalytic performance, electron paramagnetic resonance (EPR) spectroscopy was conducted on ZnS-based nanocrystals with varying Mn^2+^ doping levels, with the results shown in Figure 3a. The EPR signal intensities increase consistently with higher Mn^2+^ doping levels, indicating a rise in defect concentration as the Mn^2+^ content increases. At lower doping levels, Mn^2+^ ions predominantly occupy substitutional sites within the Zn-S lattice. However, Mn^2+^ ions are more likely to occupy interstitial sites at higher doping levels, leading to stronger Mn-Mn interactions. This is evidenced by the broadening of the EPR profiles at higher Mn^2+^ doping concentrations. While increasing Mn^2+^ doping enhances defect formation, excessive doping can destabilize the crystal lattice, ultimately diminishing piezocatalytic efficiency. As a result, the optimal piezocatalytic performance was observed at a 3% Mn^2+^ doping level.

To further elucidate the degradation mechanism, 5,5-Dimethyl-1-pyrroline N-oxide (DMPO) was employed as the spin trap to capture radicals produced within the reaction system. Figure 3b presents the EPR spectra of aqueous solutions containing DMPO and 1%, 3%, and 4% Mn^2+^-doped ZnS nanocrystals after sonication, showing a profile characteristic of ·OH radicals [36,37], and thus confirming their presence. Notably, the highest EPR signal intensity for ·OH radicals was observed in ZnS:3%Mn^2+^, consistent with its superior piezocatalytic performance. Figure 3c depicts the EPR spectra of the same Mn^2+^-doped ZnS nanocrystals/DMPO dispersions in dimethyl sulfoxide (DMSO), also after sonication. The EPR signals detected in this setup correspond to ·O_2_^−^ radicals [38], with the highest intensity detected at 3% Mn^2+^ doping. These findings indicate the formation of reactive oxygen species (ROS), such as ·OH and ·O_2_^−^, within the reaction environment. The proposed radical formation pathway during piezocatalysis is illustrated in Figure 3d. Upon sonication, the piezoelectric ZnS nanocrystals undergo tilting of band gap structure and charge separation, likely due to both electron detrapping from trap states and electron transition from the valence band to the conduction band, which drive the generation of these reactive species. Specifically, holes react with water molecules to produce hydroxyl radicals (·OH), while electrons combine with oxygen molecules to generate superoxide radicals ·O_2_^−^.

The charge separation behavior of ZnS:Mn^2+^ nanocrystals under sonication was further confirmed by more in-depth studies, e.g., modifying the surface of ZnS:3%Mn^2+^ nanocrystals with Pluronic F-127, a hole scavenger. After pre-excitation with UV light, the nanocrystals were dispersed in degassed water and subjected to sonication. If charge separation occurred, the holes would be scavenged by Pluronic F-127, and the electrons would partially convert into hydrated electrons in the solution. These hydrated electrons are known to react with oxidative species, such as hydrogen peroxide (H_2_O_2_) and hydroxyl radicals (·OH). The concentration of these oxidative species can be quantified by reacting with molecules that can change their light absorbance, for example, potassium iodide (KI) for H_2_O_2_ and salicylic acid (SA) for ·OH. In the first experiment, the UV pre-excited nanocrystals were combined with H_2_O_2_ and then sonicated. After centrifugation to remove nanocrystals, the supernatant was mixed with KI. UV-Vis spectroscopy was used to monitor the formation of triiodide $\left(I_{3}^{-}\right)$, which followed these reactions: $H_{2}O_{2}+2I^{-}\to I_{2}+2OH^{-}$ and $I^{-}+I_{2}+I_{3}^{-}$. As shown in Figure 3e, the absorbance peak of $I_{3}^{-}$ around 350 nm was significantly lower in the presence of ZnS:3%Mn^2+^ nanocrystals (1 mg/mL) compared to the control experiment without nanocrystals, indicating the consumption of H_2_O_2_ by piezo-generated electrons and, therefore, reduced formation of $I_{3}^{-}$.

In the second experiment, the reaction between SA and ·OH radicals was used to investigate charge carrier separation, as electrons can reduce hydroxyl radicals, leading to observable changes in the UV-Vis spectra. ·OH was produced through a Fenton reaction involving ferrous sulfate and H_2_O_2_ and subsequently mixed with ZnS:3%Mn^2+^ nanocrystals. The resulting oxidized product of SA, which appeared purple, exhibited an absorbance peak around 520 nm. Figure 3f shows that the absorbance decreased in the presence of ZnS:3%Mn^2+^ nanocrystals, confirming the reaction of the ·OH radicals with electrons generated from the nanocrystals. Moreover, the comparison of the absorbance with and without UV pre-excitation (Figure 3e,f) revealed that pre-excited samples had significantly lower absorbance for both $I_{3}^{-}$ and oxidized SA. This result confirms that UV pre-excitation enhances electron generation during sonication by filling trap states within the ZnS:3%Mn^2+^ nanocrystals. These findings are consistent with the piezocatalytic data discussed previously.

Control experiments were also performed to compare the absorbance of systems with and without sonication in the absence of nanocrystals. As illustrated in Figure 3e,f, sonication alone did not significantly decompose H_2_O_2_ or degrade ·OH, confirming that the observed effects were due to the presence and charge separation of the ZnS:3%Mn^2+^ nanocrystals.

**Figure 4.**
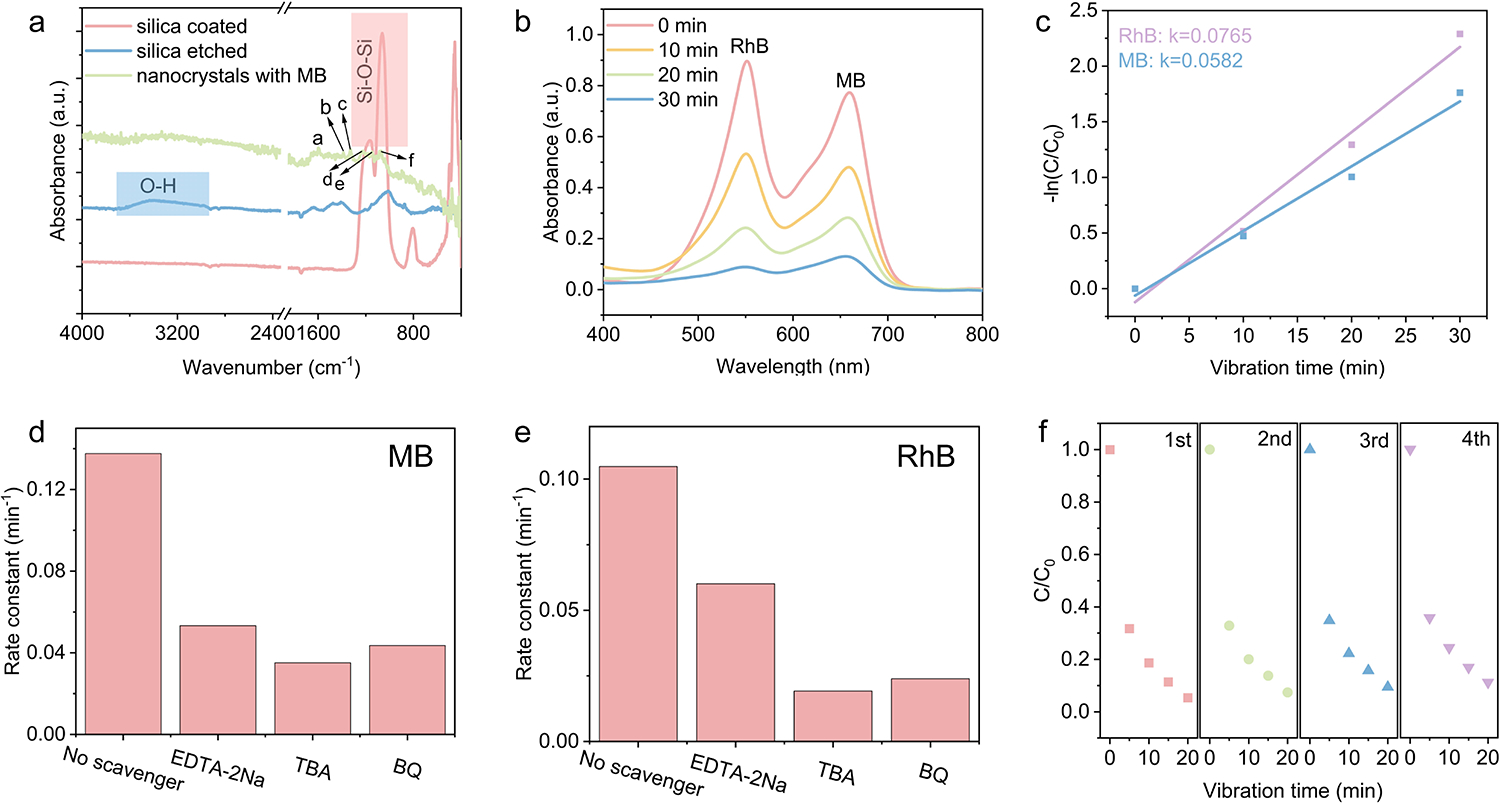
(**a**) FTIR spectra of silica-coated and silica-etched ZnS:3%Mn^2+^ nanocrystals. (**b**) The UV-Vis spectra of RhB and MB binary dye mixture during piezocatalytic degradation by ZnS:3%Mn^2+^. (**c**) The degradation rate for RhB and MB by linear fitting. (**d**) The kinetics of MB degradation with and without scavengers. (**e**) The kinetics of RhB degradation with and without scavengers. (**f**) The recyclability tests of ZnS:Mn^2+^ nanocrystal piezocatalysts.

The impact of the silica layer on the piezocatalytic performance of the nanocrystals was systematically investigated. Figure 4a shows the FTIR spectra of the ZnS:3%Mn^2+^ nanocrystals before and after removing the silica coating. For the silica-coated samples, strong Si-O-Si vibrational peaks (highlighted in the pink region) appeared around 1060 and 1160 cm^−1^. These peaks were significantly diminished after silica etching, suggesting effective removal of the silica layer. The adsorption of MB on ZnS:Mn^2+^ nanocrystals was further confirmed through FTIR analysis of the nanocrystal-MB mixture, collected after reaching adsorption-desorption equilibrium. The characteristic peaks at 1600 cm^−1^ (peak a, CH=N), 1388–1335 cm^−1^ (peak b,c, C-H), 1253 cm^−1^ (-C-N), 1153 cm^−1^ (C-N), and 1064 cm^−1^ (C-S-C) correspond to vibrations from MB, consistent with the FTIR spectrum of pure MB (Figure S13). The successful adsorption of these dyes on the nanocrystal surface is a critical factor contributing to the enhanced catalytic performance of the ZnS-based nanocatalysts.

The UV-Vis spectra of MB during piezocatalytic degradation using silica-coated ZnS:3%Mn^2+^ nanocrystals are shown in Figure S14. The degradation rate constant for silica-coated ZnS:3%Mn^2+^ was calculated to be 0.0597 min^−1^, significantly lower than that for silica-etched nanocatalysts. This result suggests that the presence of a silica layer is detrimental to the piezocatalytic degradation of organic dyes. A plausible explanation is that after undergoing high-temperature calcination, the silica layer increased its crosslinking degree and reduced its porosity [39], inhibiting effective piezocatalysis.

We also measured the piezocatalytic degradation of a binary dye mixture of MB and RhB with the same initial concentration using ZnS:3%Mn^2+^ nanocrystals. Figure 4b shows the UV-Vis spectra of the dye mixture during sonication for 30 min, with measurements taken at 10-min intervals. Figure 4c presents the degradation rates for RhB (0.0765 min^−1^) and MB (0.0582 min^−1^), respectively, as derived from linear fitting. RhB degraded faster than MB in the dye mixture, likely due to their different molecular orbital energy levels, which may facilitate electron transfer from MB to RhB [28].

To further verify the mechanism behind the piezocatalytic degradation of organic molecules, scavengers were introduced into the reaction system to assess their impact on the catalytic performance, including ethylenediaminetetraacetic acid disodium salt (EDTA-2Na) as a hole (h^+^) scavenger, tert-butyl alcohol (TBA) as a ·OH radical scavenger, and benzoquinone (BQ) as a ·O_2_^−^ radical scavenger. These scavengers were added to the reaction system containing ZnS:3%Mn^2+^ nanocrystals and organic dye molecules of MB or RhB. The UV-Vis spectra of MB during piezocatalytic degradation by ZnS:3%Mn^2+^ nanocrystals with EDTA-2Na, TBA, and BQ scavengers are shown in Figure S15a–c, respectively. The degradation kinetics were linearly fitted, yielding rate constants of 0.0533, 0.0351, and 0.0436 min^−1^ for EDTA-2Na, TBA, and BQ, respectively, as presented in Figure S15d–f. These kinetic values are summarized and compared in Figure 4d. Similarly, the UV-Vis spectra of RhB during piezocatalytic degradation in the presence of EDTA-2Na, TBA, and BQ are depicted in Figure S16a–c. The corresponding degradation rates were linearly fitted and found to be 0.0601, 0.0192, and 0.0239 min^−1^ for EDTA-2Na, TBA, and BQ, respectively, as illustrated in Figure S16d–f. These results are compared in Figure 4e. The significant reduction in degradation rates upon the addition of these scavengers supports the involvement of holes, hydroxyl radicals, and superoxide radicals in the piezocatalytic degradation process, further confirming the role of reactive oxygen species in the catalytic reaction.

The reusability of the Mn-doped ZnS piezocatalysts was evaluated through multiple catalytic cycles. As shown in Figure 4f, the relative concentration of MB (C/C_0_) was monitored over four consecutive cycles using recycled ZnS:3%Mn^2+^ nanocrystals. Only slight decline in degradation efficiency was observed after four cycles, indicating high stability in catalytic activity, possibly due to the refillable trap states and stable crystal structure of the nanocrystals. Overall, the ZnS:3%Mn^2+^ nanocrystals exhibit substantial retention of their piezocatalytic performance, underscoring their potential for practical applications where catalyst reusability is essential.

The ZnS:3%Mn^2+^ nanocrystals synthesized in this study demonstrate exceptional piezocatalytic performance, outperforming many existing piezocatalysts such as BaTiO_3_ [41], ZnO [42], metal dichalcogenides (MoS_2_, WS_2_, and WSe_2_) [43] and BiFeO_3_ [44,45], as shown in the comparison in Table 1. This enhanced performance can be primarily attributed to two key factors: the unique electron filling-detrapping mechanism enabled by UV pre-excitation and ultrasonic vibration, as well as the intrinsically high piezoelectricity of the nanocrystals. TEM images (Figures 1e and S4a) reveal a significant presence of stacking faults within the ZnS nanocrystals. These stacking faults are believed to greatly enhance the piezoelectric properties of the nanocrystals by inducing spontaneous polarization [46], consistent with the high piezoelectric coefficient (d_33_) values measured via PFM. This piezoelectric enhancement is particularly advantageous for piezocatalysis, as it facilitates effective charge separation under mechanical vibrations, thereby generating more reactive radicals for the degradation of pollutants.

We believe that the piezoelectric enhancement of ZnS:Mn^2+^ nanocrystals benefits from their dual-defect structure, which comprises both point defects resulting from Mn^2+^ doping and planar stacking faults generated during the sintering of the clusters. The point defects create numerous trap states within the bandgap, capable of storing photo-excited charge carriers that are essential for initiating subsequent piezocatalytic reactions. Meanwhile, the stacking faults likely enhance the piezoelectric effect by promoting spontaneous polarization and promote electron transport to the surface for chemical reactions [47,48]. These defects can efficiently inhibit charge carrier recombination, transforming ZnS:Mn^2+^ from a well-known luminescent material into active piezocatalysts [49–53]. We believe these two types of defects may work synergistically to not only maximize the generation of reactive oxygen species but also ensure a more sustainable and repeatable piezocatalytic process, making these nanocrystals highly promising for practical applications in environmental remediation and other catalytic fields.

## 3. Conclusion

We have successfully synthesized ZnS:Mn^2+^ nanocrystals with exceptional piezocatalytic performance for the degradation of organic pollutants. Our flexible synthesis method allows for precise tuning of the Mn^2+^ doping ratio, resulting in optimal piezocatalytic activity at 3% Mn^2+^ doping. This composition achieved a degradation rate of 0.1376 min^−1^ for methylene blue and 0.1048 min^−1^ for rhodamine B under ultrasonic vibrations. A distinctive advantage of this piezocatalyst is its significantly enhanced degradation efficiency when pre-excited with UV light prior to sonication. In-depth investigations revealed that the catalytic process is mediated by reactive oxygen species, including hydroxyl and superoxide radicals, generated under ultrasonic vibrations. The superior performance is attributed to the synergistic effects of two types of defects within the nanocrystals: Mn-induced point defects that facilitate efficient charge separation and stacking faults that enhance piezoelectricity. These combined effects substantially boost piezocatalytic activity, positioning the doped-ZnS nanocrystals as promising catalysts for environmental remediation and broader catalytic applications.

## Supplementary Material

Supporting Information

## Figures and Tables

**Table 1.** Comparison of degradation rates for various piezocatalysts under ultrasonic vibrations.

Catalyst	Dye Species	Rate Constant (min^−1^)	Reference
BaTiO_3_ nanowires	Methylene orange	~0.017	[41]
BaTiO_3_ nanocrystals	Methylene orange	0.019	
	RhB	0.009	[10]
	MB	~0.007	
(Ba,Sr)TiO_3_ nanowires	Methylene orange	0.0196	[15]
ZnO nanoparticles	MB	0.02487	[42]
MoS_2_			[43]
WS_2_	RhB	0.047	[43]
WSe_2_	RhB	0.023	[43]
BiFeO3	RhB	0.017	[44]
BiFeO_3_/PVDF-TrFE	RhB	0.0143	[45]
ZnS:3%Mn^2+^ (UV pre-excited)	MB	0.1376	This work
	RhB	0.1048	

We believe that the piezoelectric enhancement of ZnS:Mn^2+^ nanocrystals benefits from their dual-defect

## Data Availability

The data that support the findings of this study are available from the corresponding authors upon reasonable request.
