# Supplementary material for: Piezocatalytic ZnS: Mn2+ Nanocrystals for Enhanced Organic Dye Degradation
Source: Mater Interfaces. Author manuscript; Available in PMC 2025 Aug 4. (PMC12320963; doi:10.53941/mi.2024.100005)
Supplement: Supporting Information [file NIHMS2039196-supplement-Supporting_Information.pdf]

Supporting Information

Piezocatalytic ZnS: Mn^{2+} Nanocrystals for Enhanced Organic Dye Degradation

Zhongxiang Wang [1], Elizaveta Tiukalova [2], Youyi Tai [3], Miaofang Chi [2], Jin Nam [3,*] and Yadong Yin [1,*]

1 Department of Chemistry, University of California, Riverside, CA 92521, USA
2 Center for Nanophase Materials Sciences, Oak Ridge National Laboratory, Oak Ridge, TN 37831, USA
3 Department of Bioengineering, University of California, Riverside, CA 92521, USA
* Correspondence: jnam@engr.ucr.edu (J.N.); yadong.yin@ucr.edu (Y.Y.)

Received:15 October 2024; Revised: 20 November 2024; Accepted: 21 November 2024; Published: 22 November 2024

Chemicals: Zinc acetate dihydrate (>98%), rhodamine B and ferrous sulfate heptahydrate (≥99%) were purchased from Acros Organics. Cyclohexane (99.7%) and potassium iodide (KI, 99%) was purchased from Alfa Aesar. Manganese acetate tetrahydrate (≥99%) was purchased from STREM Chemicals. 5,5-Dimethyl-1-pyrroline N-oxide (DMPO) were purchased from TCI chemicals. Oleylamine (technical grade, 70%), Cetyltrimethylammonium bromide (CTAB, ≥99%), Tetraethyl orthosilicate (TEOS, ≥99%), methylene blue, Phosphate Buffered Saline (PBS) buffer (pH 7.4, 100mM), Pluronic F-127 and ethylenediaminetetraacetic acid disodium salt dihydrate (EDTA-2Na, ≥99%) were purchased from Sigma Aldrich. Ammonia (28 wt.% NH_3), absolute ethanol (200 proof), hydrogen peroxide (H_2O_2, 30%) and sodium hydroxide (NaOH) were purchased from Fisher Scientific. Tert-butyl alcohol (TBA, ≥99%), benzoquinone (99%) was purchased from Thermo Scientific. Salicylic acid (SA, ACS reagent grade) was purchased from MP Biomaterials.

ZnS: x at.% Mn^{2+} (x = 0, 0.5, 1, 2, 3, 4) quantum dot synthesis: ZnS:Mn^{2+} quantum dots were synthesized using a method adapted from the literature [29]. In this synthesis, a total of 20 mmol of zinc acetate dihydrate and manganese acetate tetrahydrate were combined in a 250-mL three-neck round bottom flask containing 80 mL of oleylamine. The amount of manganese acetate was determined by the nominal atomic ratios of Mn^{2+}. This mixture was initially bubbled with nitrogen at 170 °C for one hour to facilitate the formation of a complex between the metal ions and oleylamine, while simultaneously removing water and acetic acid molecules. Subsequently, the temperature was increased to 300 °C under a continuous flow of nitrogen. At this target temperature, 10 mL of 1-dodecanethiol (serving as the sulfur source, 40 mmol) was injected into the mixture. The temperature was maintained for 3 h to ensure a complete reaction between metal acetates and 1-dodecanethiol. Following the reaction, the quantum dots were precipitated by adding 160 mL of ethanol and then isolated using centrifugation at 9000 rpm for 10 min. The precipitate was redispersed in hexanes and re-precipitated with ethanol, followed by another round of centrifugation. The final product was transferred to a vacuum chamber and dried at room temperature to obtain the purified ZnS:Mn^{2+} quantum dots. The dried quantum dots were dispersed in cyclohexane in a concentration of 100 mg/mL as stock solution.

Self-assembly of ZnS:Mn^{2+} quantum dots: Typically, 2-mL ZnS: x at.% Mn^{2+} quantum dot stock solution was mixed with 18 mL of cyclohexane to obtain a solution in a concentration of 10 mg/mL. The quantum dot solution was then mixed with 200-mL CTAB aqueous solution (5.6 mg/mL) and sonicated for 1.5 min using a sonication probe (U.S. Solid Ultrasound Processor, model: USS-HLUH, probe diameter: 30 mm, working power: 450 W (25% of total 1800 W), frequency: 20 kHz) to obtain a uniform milky oil-in-water emulsion. Then the emulsion was heated at 70 °C for 4 h in an oil bath to evaporate the cyclohexane. The assemblies were collected by centrifugation at 10000 rpm for 10 min.

Silica coating: The assemblies were coated with a silica layer using the Stöber method [32]. Initially, the assemblies were re-dispersed in 15 mL of water, to which 100 mL of ethanol was added. Subsequently, 5 mL of ammonia and 400 µL of tetraethyl orthosilicate (TEOS) were introduced. The mixture underwent vigorous stirring for 25 min to ensure uniform silica growth. The silica-coated assemblies were then isolated by centrifugation at 9000 rpm for 5 min, re-dispersed in ethanol, and centrifuged again to collect the precipitates. Finally, the collected material was dried in a vacuum oven at 60 °C for 3 h.

Calcination: The silica-coated assemblies were calcined at 1050 °C for 1.5h in a tube furnace (Thermo Scientific Linderberg Blue M 1100 °C tube furnace) under nitrogen flow to obtain silica-coated nanocrystals in wurtzite phase.

Copyright: © 2024 by the authors. This is an open access article under the terms and conditions of the Creative Commons Attribution (CC BY) license (https://creativecommons.org/licenses/by/4.0/).
Publisher's Note: Scilight stays neutral with regard to jurisdictional claims in published maps and institutional affiliations

Silica etching: The silica coating on the surface was removed via NaOH treatment at an elevated temperature. Specifically, 100 mg of calcined silica-coated nanocrystals were dispersed in a 1 M NaOH solution, which was prepared by dissolving 800 mg of NaOH (20 mmol) in 20 mL of water. This dispersion was continuously stirred at 70 °C for 8 h within an oil bath. Subsequently, the nanocrystals were isolated through repeated centrifugation at 8000 rpm for 5 min. Finally, the nanocrystals were dried in a vacuum oven at 60 °C for 3 h.

Piezocatalysis: In a typical experiment, 20 mg of ZnS:Mn^{2+} nanocrystals were uniformly dispersed in 10 mL of water and combined with 10 mL of a 20 ppm dye stock solution. The mixture was stirred in darkness for 30 min to establish adsorption-desorption equilibrium. Subsequently, the solution was subjected to sonication at 40 kHz in a temperature-controlled sonication reactor, maintained at 22 °C. The piezocatalytic reaction proceeded in the absence of light. UV-Vis spectra were recorded periodically at 5 or 10-min intervals post-sonication. After the sonication process, a 2 mL sample was collected, and the dye was separated from the nanoparticles through centrifugation at 14,000 rpm for 2 min. The resulting supernatant was then transferred to a cuvette (3.5 mL, pathlength: 1cm), and the UV-Vis spectrum was measured using a spectrophotometer.

Scavenging of active species: EDTA-2Na (h^+ scavenger), tert-butanol (·OH hydroxyl radical scavenger), and 1, 4-benzoquinone ($\cdot O_2^-$ radical scavenger) were added into the dye-catalyst suspension, respectively, in a concentration of 1 mmol/L. The same experiment procedure was conducted to investigate the role of active species.

Detection of Active Species: ·OH radicals were generated by dispersing 5 mg ZnS:Mn^{2+} nanocrystals s in 5 mL water and sonicating for 10 min. The ·OH radicals were trapped by mixing 200 μL of ZnS nanoparticle dispersion with 200 μL 400 mM DMPO aqueous solution and then sonicating for another 5 min. A capillary tube was dipped into the mixture and the solution was taken out for EPR measurement. $\cdot O_2^-$ radicals were generated by sonicating ZnS:Mn^{2+} nanocrystals in DMSO in the same concentration, and then trapped by using 400 mM DMPO/DMSO solution.

Pluronic F-127 surface modification: Pluronic F-127 and ZnS:Mn^{2+} nanocrystals in a mass ratio of 20:1 were sonicated in aqueous solution for 30 min. The nanocrystals were collected by repeated centrifugation and the supernatant containing extra Pluronic F-127 was trashed.

Evidence of charge separation by KI-H_2O_2 system: The Pluronic F-127 modified ZnS:3%Mn^{2+} nanocrystals were dispersed in water in a concentration of 1 mg/mL. 0.5 mL of dispersion was pre-excited by 365-nm UV light. The pre-excited dispersion was then mixed with 0.5 mL of 10 mM PBS and 0.5 mL of 100 mM H_2O_2 solution. The mixture was sonicated for 30 min and the nanocrystals were separated by centrifugation (14,000 rpm, 90 s). 0.8 mL of supernatant was mixed with 0.8 mL of 1M KI solution. The solution mixture was incubated at room temperature for 5 min and measured by the UV-Vis spectrometer. The control experiment without nanocrystals was conducted by adding pure water instead of nanocrystal dispersion.

Evidence of charge separation by SA-·OH system: The ·OH radicals were generated by Fenton reaction involving 1 mL of 20 mM ferrous sulfate and 1 mL of 20 mM H_2O_2 solution. Then 1 mL of ZnS:3%Mn^{2+} aqueous dispersion in a concentration of 1mg/mL was pre-excited by UV light and mixed with the above solution containing ·OH radicals. The mixture was sonicated for 30 min and centrifuged at 14000 rpm for 90s. 0.8 mL of the supernatant was mixed with 0.8 mL of SA solution in a concentration of 1 mM. The solution mixture was measured by a UV-Vis spectrometer. The control experiment without nanocrystals was conducted by adding pure water instead of nanocrystal dispersion.

Optical spectroscopy: UV-Vis absorption spectra were obtained using an Agilent Cary 60 UV-Vis spectrometer with a step size of 600 nm/min. Fourier transform infrared spectroscopy (FTIR) spectra were obtained on a Nicolet iS50 FTIR spectrometer in a Attenuated Total Reflectance (ATR) mode. The wavenumber range is 4000–400 cm^{-1}.

X-ray diffraction (XRD): The XRD was measured on a PANalytical Empyrean Series 2 X-ray diffractometer. Step size: 0.10°. Target: copper. λ(Cu K_α): 1.5406 Å.

Transmission electron microscopy (TEM): TEM was performed on a Thermo Fisher Scientific Talos L120C microscope, equipped with a single-tilt stage and operated at an accelerating voltage of 120 kV. High-angle annular dark-field (HAADF) imaging andSTEM-energy dispersive X-ray spectroscopy (EDS) mapping were performed using a JEOL-ARM200F (NEOARM) microscope, operated at an accelerating voltage of 200 kV. This instrument was fitted with dual 100 mm^2 JEOL solid-state EDS detectors, providing a large total solid angle for efficient signal collection.

Electron spin resonance (ESR) spectroscopy: ESR measurements were performed using a Bruker ESR5000 Benchtop instrument. The parameters for the analysis were as follows: magnetic field range from 300 to 370 mT, sweep time of 60 s, modulation amplitude of 0.2 mT, and frequency modulation of 100 kHz, with a resonance frequency of 9.4 GHz. Samples, either in dry powder form or in solution, were placed in a quartz tube (6 mm outer diameter) to a height of 1.5 cm. The tube was then inserted into the ESR instrument for analysis.

Piezoresponse force microscopy (PFM): PFM was conducted using an atomic force microscope (AFM, MFP-3D, Asylum Research) operated in air-contact mode to identify and analyze individual nanoparticles. Once the nanoparticles were characterized, the PFM mode was engaged for detailed single-point measurements. A triangular-square voltage bias was applied directly to the nanoparticles via the AFM cantilever (AC240TM, Asylum Research), allowing for the detection of piezoelectric displacements in response to the electrical bias. To ensure data reliability, at least three independent nanoparticles were analyzed, with multiple measurements recorded along the length of each nanoparticle under each experimental condition.

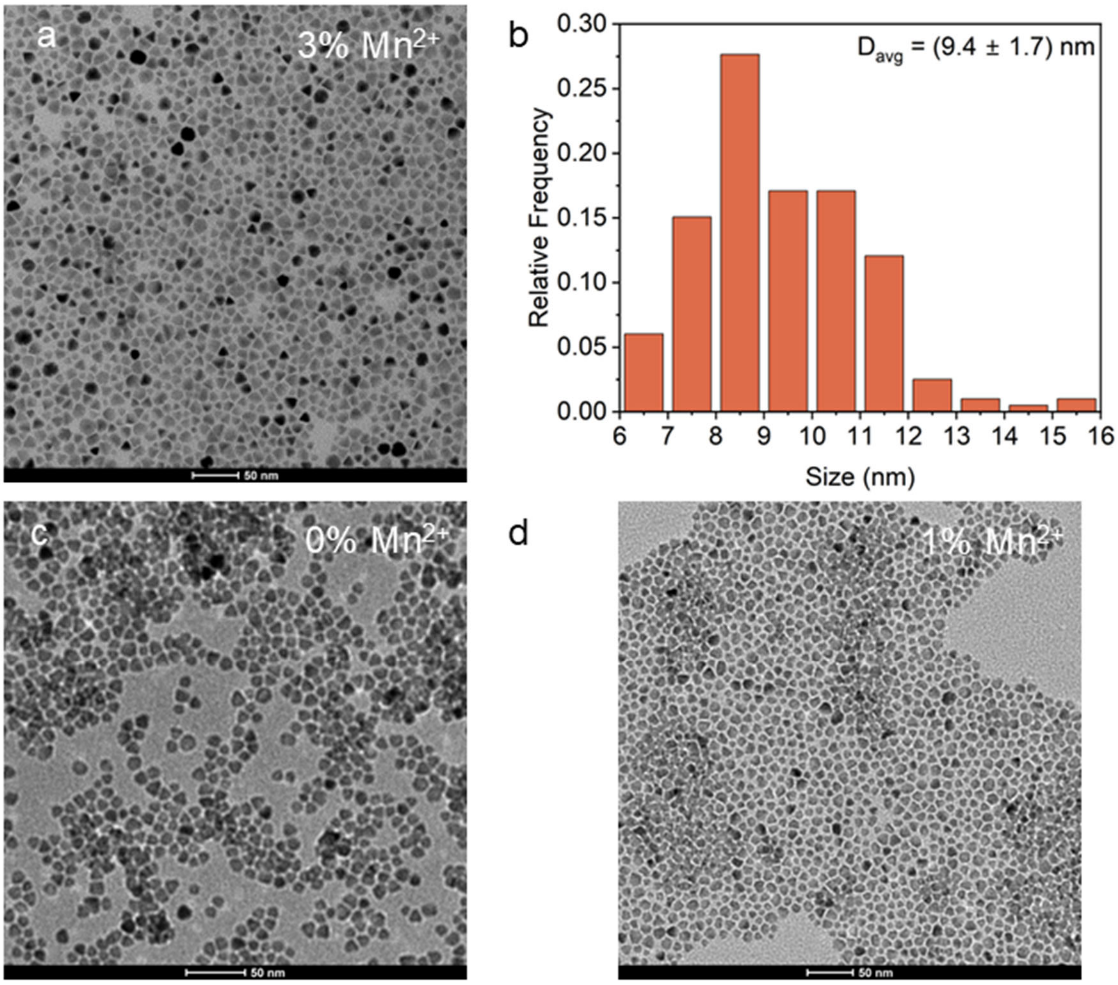

Figure S1. (**a**) The TEM image of ZnS:3%Mn^{2+} QDs; (**b**) The particle size distribution of ZnS:3%Mn^{2+} QDs; (**c**) The TEM image of ZnS QDs; (**d**) The TEM image of ZnS:1%Mn^{2+} QDs.

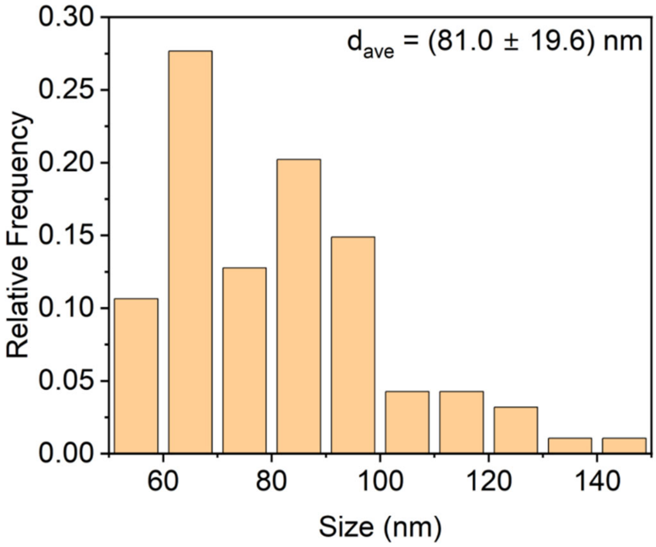

Figure S2. The size distribution of ZnS:3%Mn^{2+} QD assemblies.

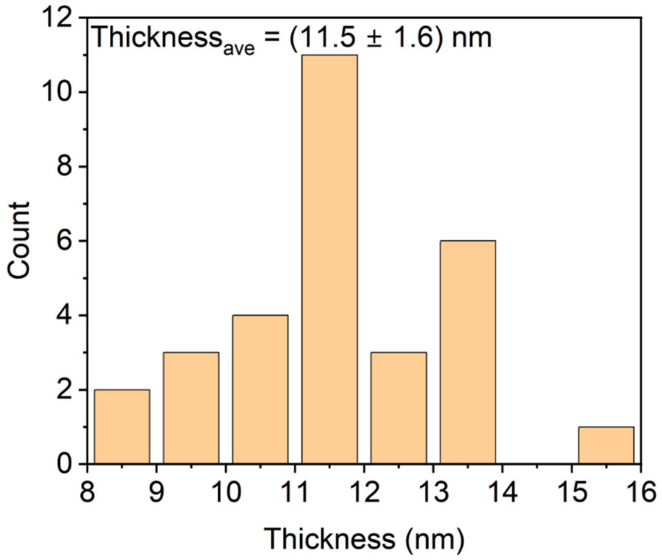

Figure S3. The thickness distribution of the silica layer around the QD assemblies.

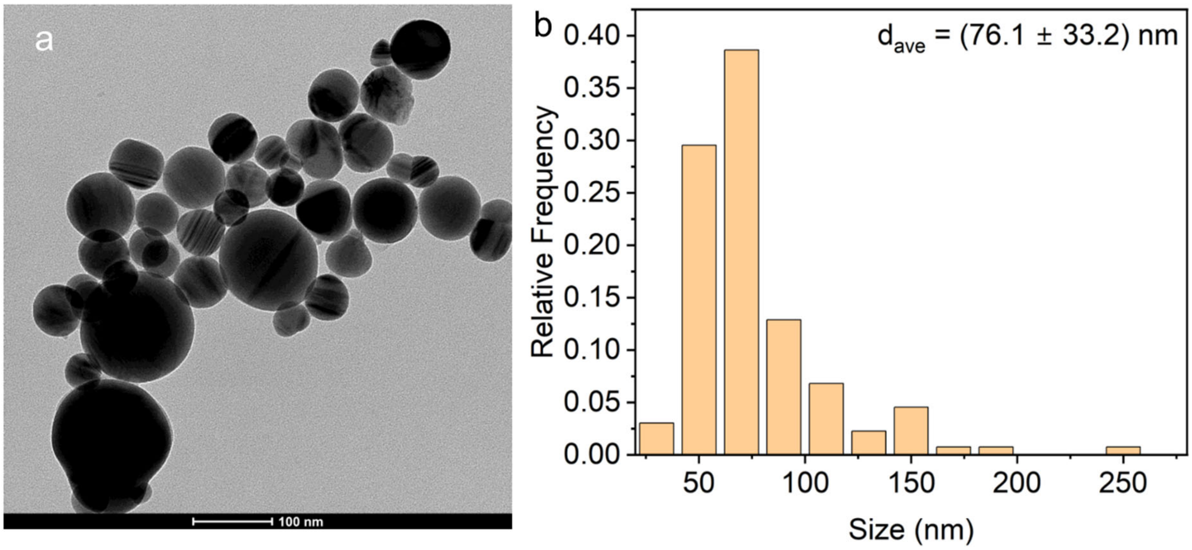

Figure S4. (**a**) The TEM image of silica-etched ZnS:3%Mn^{2+} nanocrystals; (**b**) The particle size distribution of silica-etched ZnS:3%Mn^{2+} nanocrystals.

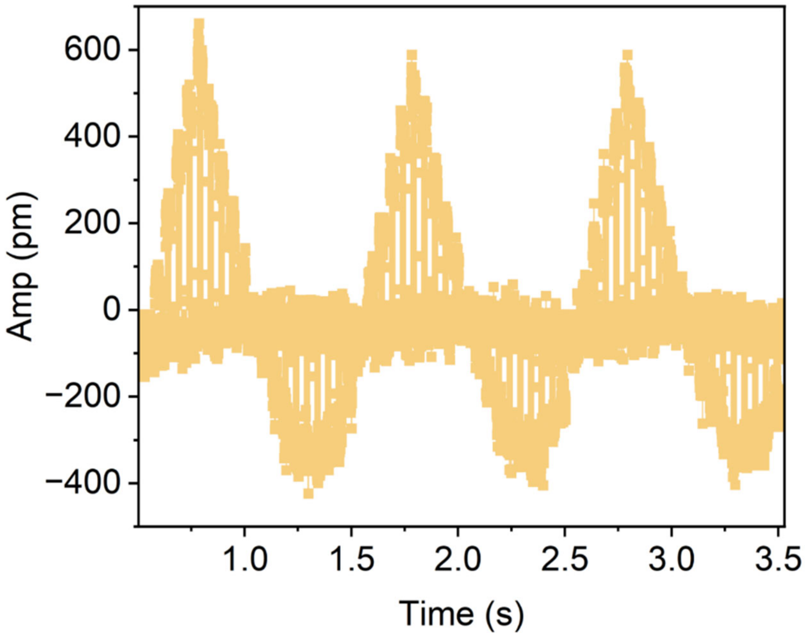

Figure S5. The PFM response amplitude of ZnS:3%Mn^{2+} nanocrystals under the voltage of 3V.

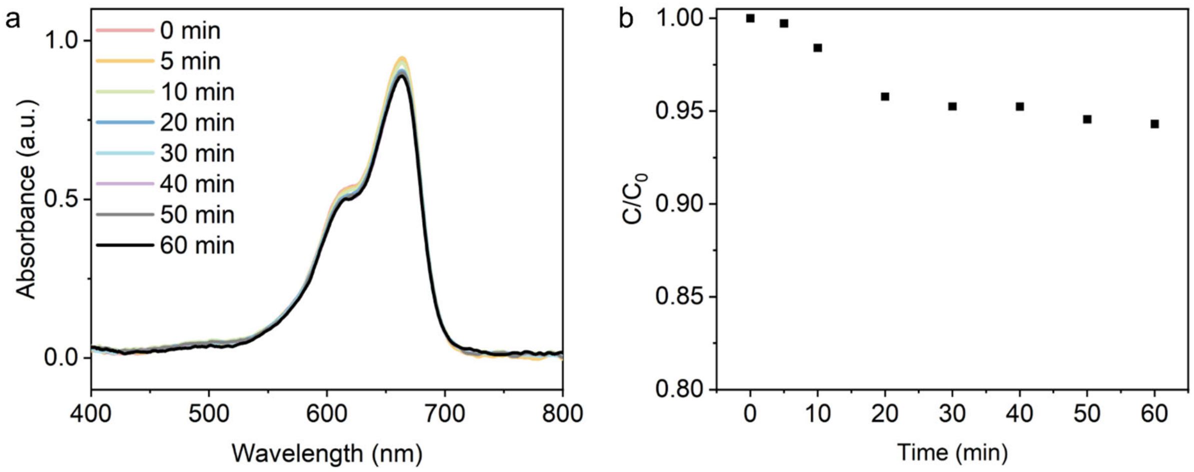

Figure S6. (**a**) The UV-Vis spectra of MB after stirring with ZnS:3%Mn^{2+} nanocrystals for different durations; (**b**) The concentration ratio (C/C_0) during the stirring.

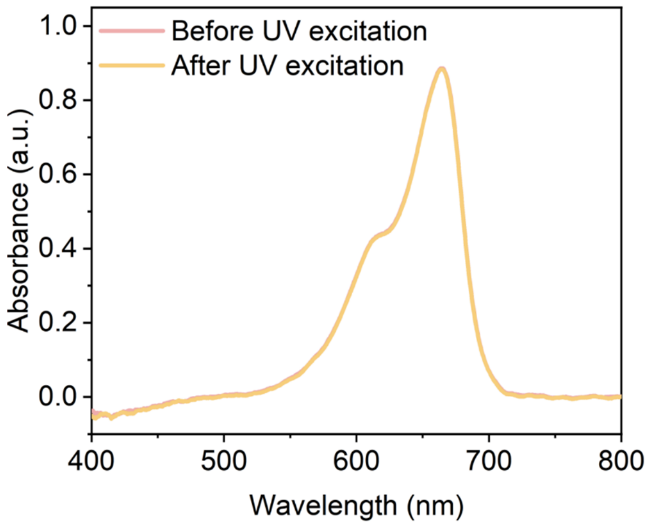

Figure S7. The UV-Vis spectra of MB solution before and after exposing the MB-nanocrystal mixture to 365-nm UV light for 1 min.

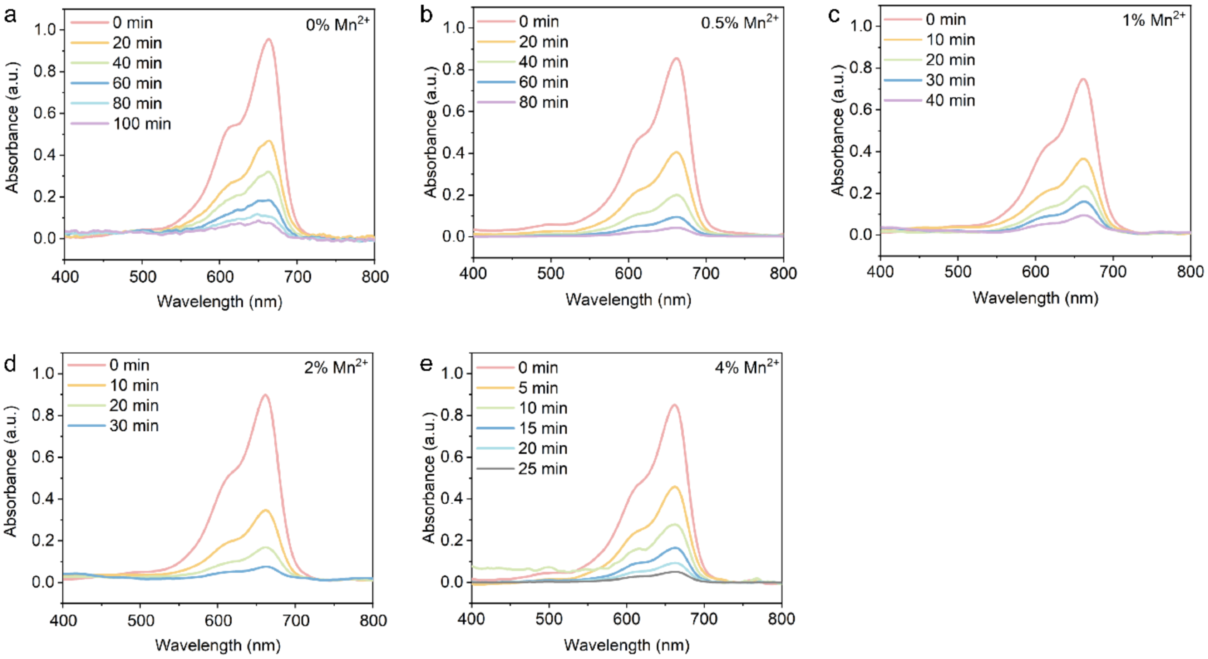

Figure S8. The UV-Vis spectra of MB during piezocatalytic degradation using 0%Mn^{2+} (**a**), 0.5%Mn^{2+} (**b**), 1%Mn^{2+} (**c**), 2%Mn^{2+} (**d**), 4%Mn^{2+} (**e**).

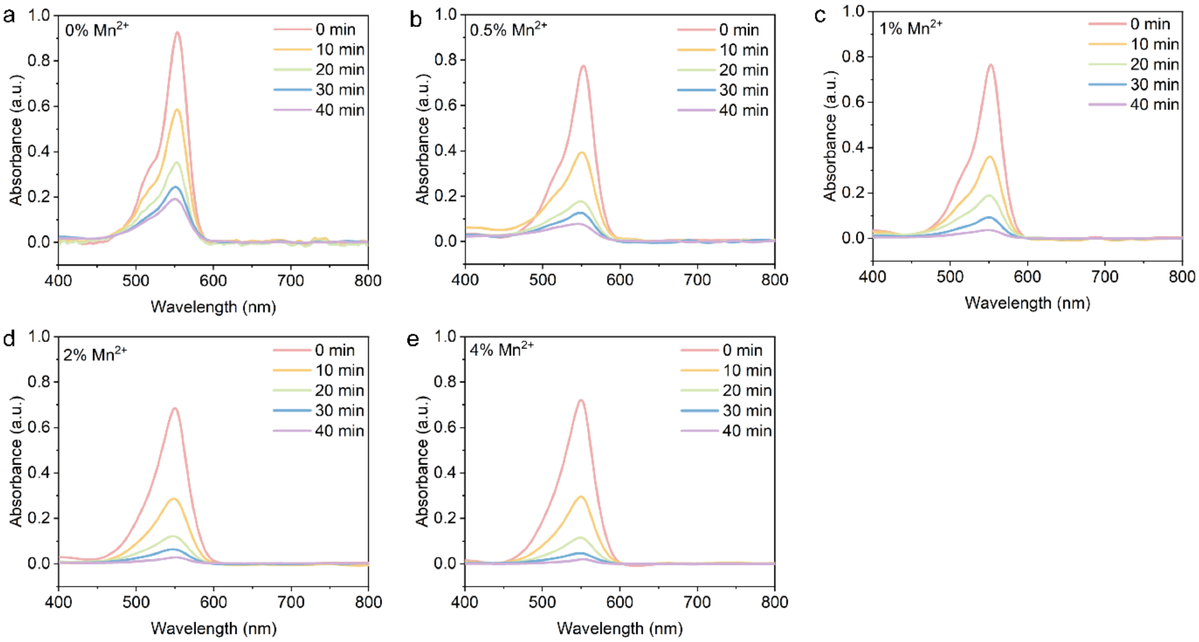

Figure S9. The UV-Vis spectra of RhB during piezocatalytic degradation using 0%Mn^{2+} (**a**), 0.5%Mn^{2+} (**b**), 1%Mn^{2+} (**c**), 2%Mn^{2+} (**d**), 4%Mn^{2+} (**e**).

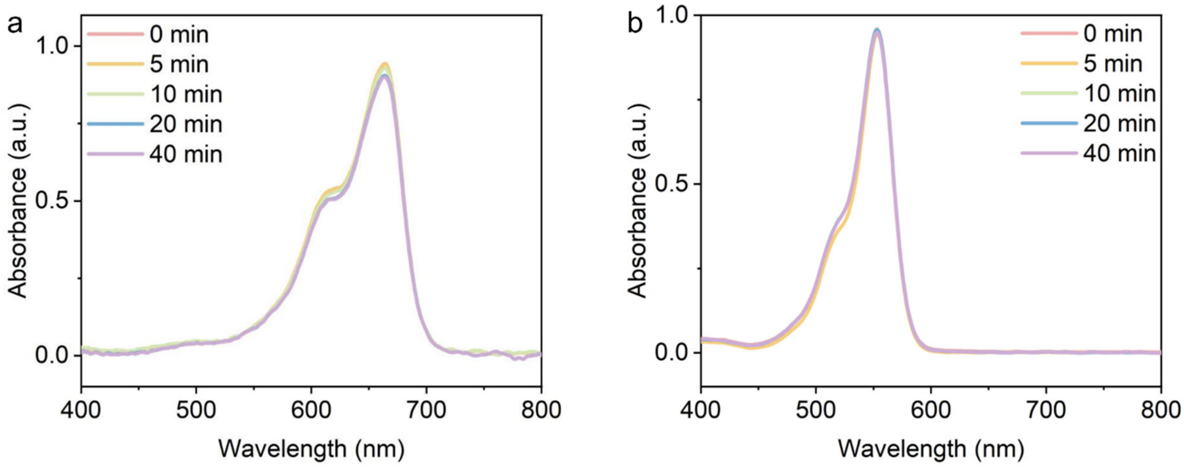

Figure S10. The UV-Vis spectra of MB (**a**) and RhB (**b**) during sonication for up to 40 min.

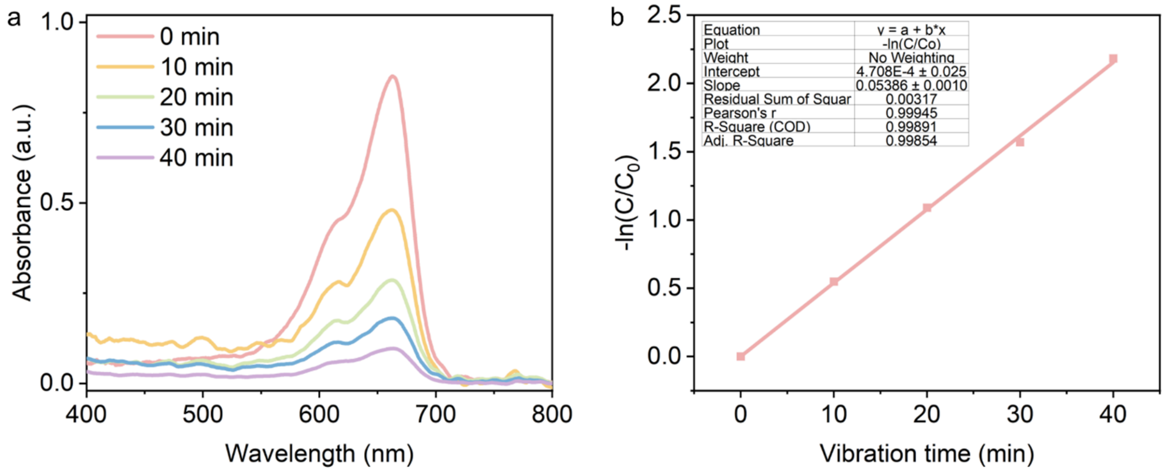

Figure S11. (**a**) The UV-Vis spectra of MB during degradation without UV pre-excitation; (**b**) The corresponding linear fitting of -ln(C/C_0) vs. vibration time plot.

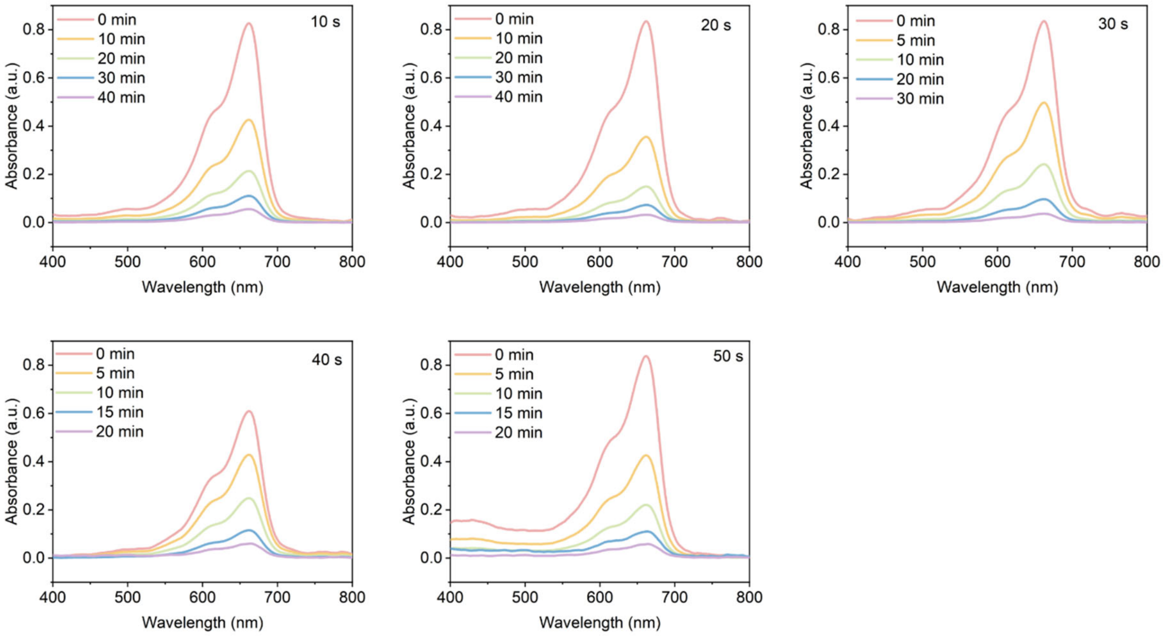

Figure S12. The UV-Vis spectra of MB during piezocatalytic degradation with UV pre-excitation for different durations.

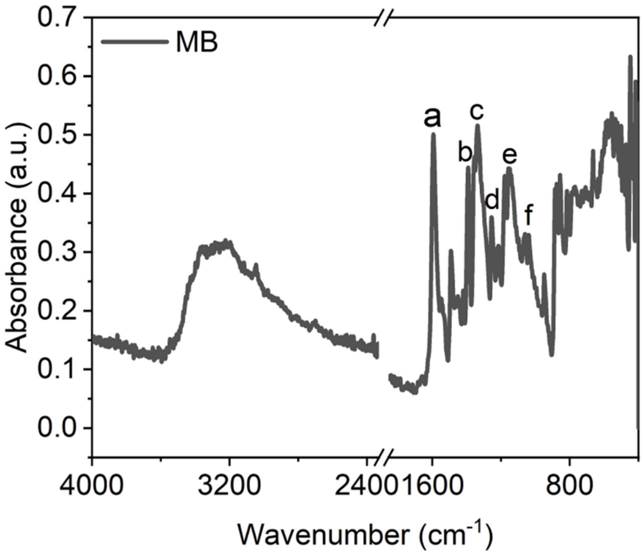

Figure S13. The FTIR spectrum of pure MB. The peaks labeled as (**a**): 1600 cm^{-1} (CH=N); (**b**,**c**): 1388–1335 cm^{-1} (C-H); (**d**): 1253 cm^{-1} (-C-N); (**e**): 1153 cm^{-1} (C-N); (**f**): 1064 cm^{-1} (C-S-C).

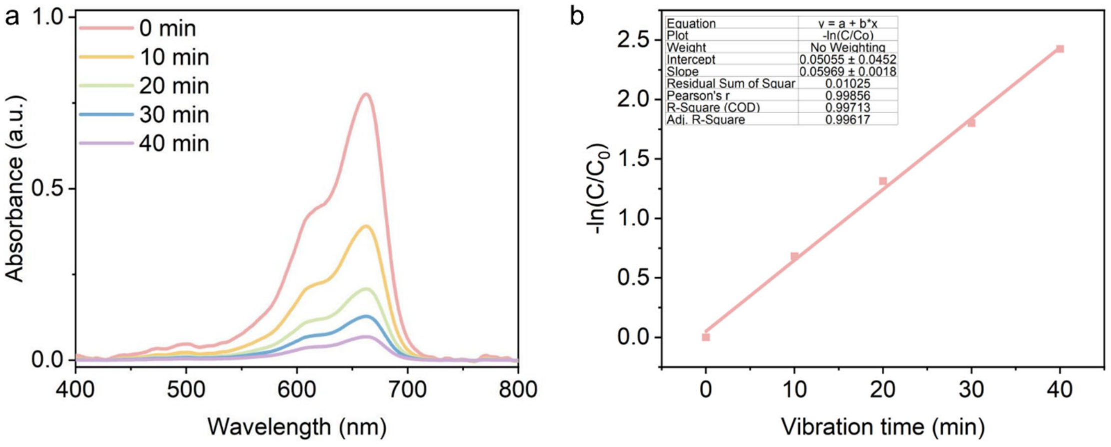

Figure S14. The UV-Vis spectra of MB during piezocatalytic degradation with silica coating around the ZnS:3%Mn^{2+} nanocrystals.

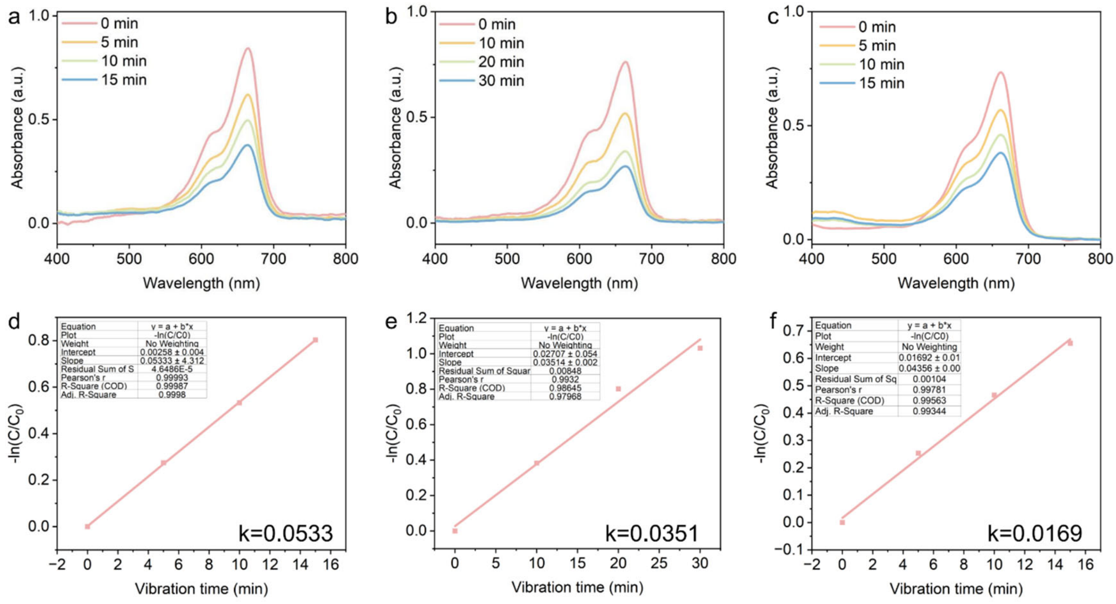

Figure S15. The UV-Vis spectra of MB during piezocatalytic degradation with the addition of scavengers: EDTA-2Na (**a**), TBA (**b**), BQ (**c**). The linear fitting of -ln(C/C_0) vs. vibration time plot for MB during piezocatalytic degradation with the addition of scavengers: EDTA-2Na (**d**), TBA (**e**), BQ (**f**).

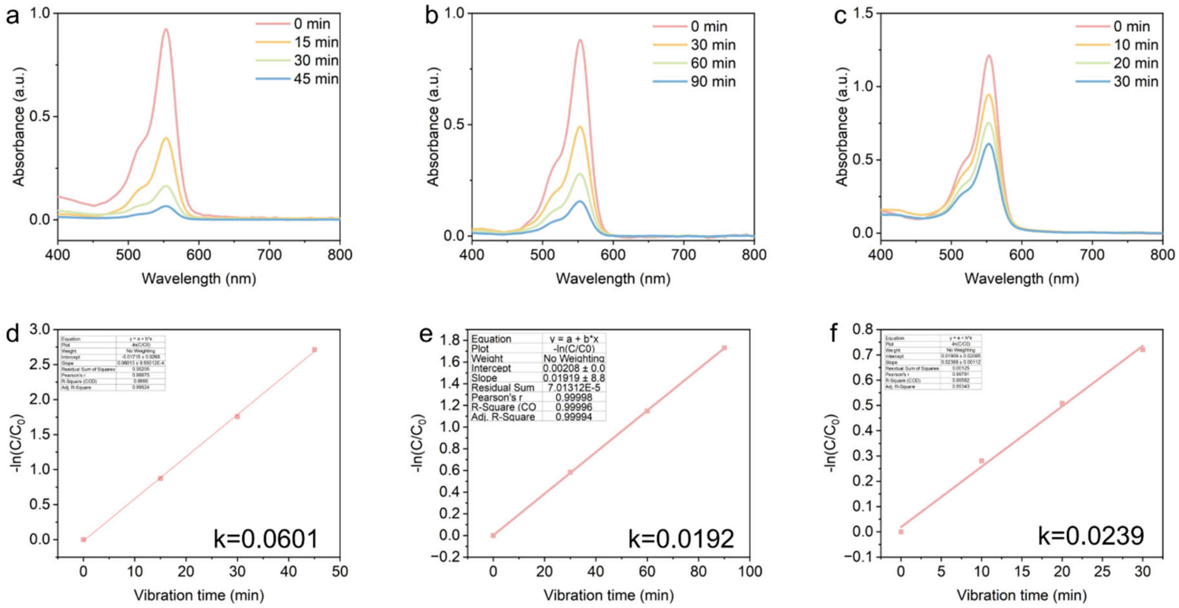

Figure S16. The UV-Vis spectra of RhB during piezocatalytic degradation with the addition of scavengers: EDTA-2Na (**a**), TBA (**b**), BQ (**c**). The linear fitting of -ln(C/C_0) vs. vibration time plot for RhB during piezocatalytic degradation with the addition of scavengers: EDTA-2Na (**d**), TBA (**e**), BQ (**f**).
